# Differences in Physicians’ Ratings of Work Stressors and Resources Associated With Digital Transformation: Cross-Sectional Study

**DOI:** 10.2196/49581

**Published:** 2024-06-17

**Authors:** Magdalena Katharina Wekenborg, Katharina Förster, Florian Schweden, Robin Weidemann, Felix von Bechtolsheim, Clemens Kirschbaum, Jürgen Weitz, Beate Ditzen

**Affiliations:** 1 Chair of Biopsychology Faculty of Psychology TU Dresden Dresden Germany; 2 Else Kröner Fresenius Center for Digital Health Faculty of Medicine and University Hospital Carl Gustav Carus TU Dresden Dresden Germany; 3 Clinical Psychology and Behavioural Neuroscience Faculty of Psychology TU Dresden Dresden Germany; 4 Institute for Work Design and Organizational Development INAGO Hamburg Germany; 5 Clinic of Internal Medicine Krankenhaus St. Joseph-Stift Dresden Germany; 6 Department of Gastrointestinal, Thoracic, and Vascular Surgery Faculty of Medicine and University Hospital Carl Gustav Carus TU Dresden Dresden Germany; 7 Center for Tactile Internet with Human-in-the-Loop (CeTI) TU Dresden Dresden Germany; 8 Institute of Medical Psychology Heidelberg University Heidelberg Germany

**Keywords:** physicians, digital transformation, chronic stress, hair cortisol concentration, work stressors, work resources

## Abstract

**Background:**

The emergence of the COVID-19 pandemic rapidly accelerated the need and implementation of digital innovations, especially in medicine.

**Objective:**

To gain a better understanding of the stress associated with digital transformation in physicians, this study aims to identify working conditions that are stress relevant for physicians and differ in dependence on digital transformation. In addition, we examined the potential role of individual characteristics (ie, age, gender, and actual implementation of a digital innovation within the last 3 years) in digitalization-associated differences in these working conditions.

**Methods:**

Cross-sectional web-based questionnaire data of 268 physicians (mean age 40.9, SD 12.3 y; n=150, 56% women) in Germany were analyzed. Physicians rated their chronic stress level and 11 relevant working conditions (ie, work stressors such as time pressure and work resources such as influence on sequence) both before and after either a fictional or real implementation of a relevant digital transformation at their workplace. In addition, a subsample of individuals (60; n=33, 55% women) submitted self-collected hair samples for cortisol analysis.

**Results:**

The stress relevance of the selected working conditions was confirmed by significant correlations with self-rated chronic stress and hair cortisol levels (hair F) within the sample, all of them in the expected direction (*P* values between .01 and <.001). Multilevel modeling revealed significant differences associated with digital transformation in the rating of 8 (73%) out of 11 working conditions. More precisely, digital transformation was associated with potentially stress-enhancing effects in 6 working conditions (ie, *influence on procedures* and *complexity of tasks*) and stress-reducing effects in 2 other working conditions (ie, *perceived workload* and *time pressure*). Younger individuals, women, and individuals whose workplaces have implemented digital innovations tended to perceive digitalization-related differences in working conditions as rather stress-reducing.

**Conclusions:**

Our study lays the foundation for future hypothesis-based longitudinal research by identifying those working conditions that are stress relevant for physicians and prone to differ as a function of digital transformation and individual characteristics.

## Introduction

### Background

The COVID-19 pandemic convincingly demonstrated the urgent need for employable physicians for the functionality of many aspects of society. Given this crucial role of physicians in society, the widespread prevalence of chronic stress among them [1] is alarming. Chronic stress is experienced when perceived resources are enduringly outweighed by demands [2]. Keeping in mind that chronic stress and its sequelae (ie, burnout symptoms) have been associated with risks for patients (eg, heightened risk for medical errors [3]), as well as a significant increase in rates of sickness absence and incapacity for work [4], chronic stress poses a serious threat to the health and employability of physicians [5].

In theory, the increasing digital transformation of the health system could help to reduce the chronic stress of physicians because the potential of improvement of working conditions of health care professionals by IT has been emphasized repeatedly [6]. Thereby, digital transformation defined as an automatization of tasks [7] can be distinguished from digitization (ie, technical process of converting analog signals into a digital form [7,8]) and digitalization (ie, the process of adopting and using this technology in broader contexts [9]). As described in a review by Topol [10], the importance of top digital health care technologies, namely, digital medicine (eg, telemedicine), genomics (eg, reading the genome), artificial intelligence (AI), and robotics (eg, automated image interpretation using AI), for the work of physicians will increase significantly within the next decades.

Indeed, theoretically, digital transformation offers a variety of ways to reduce workplace stress for physicians, including simplifying time-consuming bureaucracy and relieving certain tasks and responsibilities through digital decision-making tools [6]. Empirically, there is evidence for the stress-reducing effects of digital innovations, for example, with respect to robot-assisted surgery on surgeons’ stress load [11] and mental effort and workload [12].

However, the few existing empirical studies do not consistently support the stress-buffering effects of digital transformation on perceived stress in physicians. Particularly, electronic health records (EHRs) and comparable information systems have been implicated as a factor that might enhance physicians’ chronic stress, both in cross-sectional [13-15] and longitudinal studies [16].

Although EHRs receive significant attention, research indicates that increased use of other digital technologies, such as new surgical technologies in operating rooms [17] and telemedicine [18], may also contribute to chronic stress among physicians. These findings implicate that very different types of digital transformation can have stress-enhancing effects for the user.

One of the most established models to explain the development of stress as a consequence of digital transformation is the technostress model, which was originally introduced by Brod [19]. On the basis of the transactional model of stress and health [2], Brod [19] defined technostress as the result of an inadequate ability to cope with the requirements of the use of computer technology. More precisely, he defined the following 5 components crucially related to the development of technostress, namely, techno-invasion (employee can be contacted at any time), techno-overload (technology forces one to work harder), techno-complexity (complexity requires learning efforts), techno-insecurity (one is afraid of losing one’s job because of technology), and techno-uncertainty (continuous changes requires constant relearning).

The technostress model by Brod [19] has undoubtedly made a decisive contribution to the description and study of the phenomenon. However, its further development is highly relevant for a better understanding of the process by which digital transformation affects stress. Although there have been important expansions to the original model (eg, by Gimpel et al [20] and Ragu-Nathan et al [21]), empirical evidence on which particular stress-relevant working conditions mediate the association between digital transformation and enhanced stress is still lacking. Identifying the working conditions that contribute to physician stress and are susceptible to change would be highly relevant for monitoring and designing health care digitalization.

When it comes to the selection of these stress-relevant working conditions, different theoretical conceptualizations and empirical evidence exist [22-26]. Thereby, the working conditions identified by Rau and Buyken [27] within a meta-analysis appear to be especially suited with regard to the aim of this study, as they have been shown to be relevant in *physicians* [27,28]. The working conditions identified by this meta-analysis can be roughly subdivided into *work resources* potentially reducing stress load at work (eg, learning new skills and job control) and *work stressors* potentially enhancing stress load at work (eg, time pressure).

### Objectives

To complement existing models on technostress in physicians and enabling future hypothesis-based research, this study set out to examine (1) which of those working conditions identified by Rau and Buyken [27] are stress relevant for physicians and (2) which differ as a function of digital transformation. Thereby, in contrast to the prevailing approach in prior research, our study acknowledges stress as a biopsychological phenomenon. We do this by operationalizing stress using both self-report measures as well as a biological marker of stress, namely, hair cortisol concentrations (hair F). Shortly summarized, hair F has been shown to be a valid and solid index of the functional status of one of the central stress pathways of the body, the hypothalamus-pituitary-adrenal (HPA) axis, with high intraindividual stability and test-retest reliability, which, in contrast to other forms of cortisol quantification, represents an aggregated measure of chronic stress over a period of months [29].

To answer these research questions, the following hypotheses are tested:

- *H1*: The selected working conditions are associated with psychological and physiological markers of chronic stress in physicians. More precisely, *work resources* (ie, *influence on sequence of activities; influence on workload and procedures; learning new skills; use of knowledge, skills, and abilities; visibility of task accomplishment*; and *consideration of employee input*) are associated with reduced levels of chronic stress in physicians, and *work stressors* (ie, *workload, time pressure, excessive complexity of tasks, excessive demands on concentration*, and *interruptions of workflow*) are associated with enhanced levels of chronic stress in physicians..
- *H2*: The ratings of the selected working conditions carried out by physicians differ as a function of digital transformation
  - *H2.1*: Age and gender influence differences in these ratings.
  - *H2.2*: Finally, we were interested in how actual experiences with digital transformation at the workplace might moderate differences in the ratings of these working conditions before and after digital transformation.

## Methods

### Recruitment

Physicians were recruited throughout Germany via social networks, web-based platforms, and medical associations with a special focus on the University Hospital Dresden. Practicing physicians from all disciplines were included in the study.

A total of 437 participants started the web-based questionnaire. Of the 437 participants, 271 (62%) completed the questionnaire. Of these 271 participants, 3 (1.1%) defined their gender as “diverse.” They were not included in the analyses of the core sample because gender was included as a control variable in all subsequent analyses, and this number is too small for statistical processing, leaving a final sample of 268 participants (core sample: mean age 40.79, SD 12.3 y; n=150, 56% women). Hair samples were available from 24% (60/268) of these participants (subsample). Sample characteristics of the core sample as well as the subsample that provided hair samples are presented in Table 1.

**Table 1:** Characteristics of the core sample and the subsample.

Characteristic		Core sample (N=268)	Subsample (n=60)
Age (y), mean (SD)		40.8 (12.3)	40.5 (12.1)
Work experience (y), mean (SD)		13.98 (12.4)	13.4 (12.3)
Gender (women), n (%)		150 (56)	33 (55)
**Specialty^a^, n (%)**			
	Internal medicine	79 (29.5)	16 (27)
	General medicine	23 (8.6)	3 (5)
	Anesthesiology	21 (7.8)	6 (10)
	Surgery	20 (7.5)	3 (5)
	Psychiatry	17 (6.3)	7 (12)
	Pediatrics	15 (5.6)	6 (10)
PSS-4^b^, mean (SD)		6.3 (2.9)	6.1 (3)
Hair F^c^, mean (SD)		6.3 (5)	6.3 (5)

^a^Most frequently mentioned specialties.

^b^PSS-4: Perceived Stress Scale 4.

^c^Hair F: hair cortisol concentration.

The most commonly reported medical specialty was by far internal medicine, followed by general medicine, anesthesiology, surgery, psychiatry, and pediatrics.

**Figure 1:**
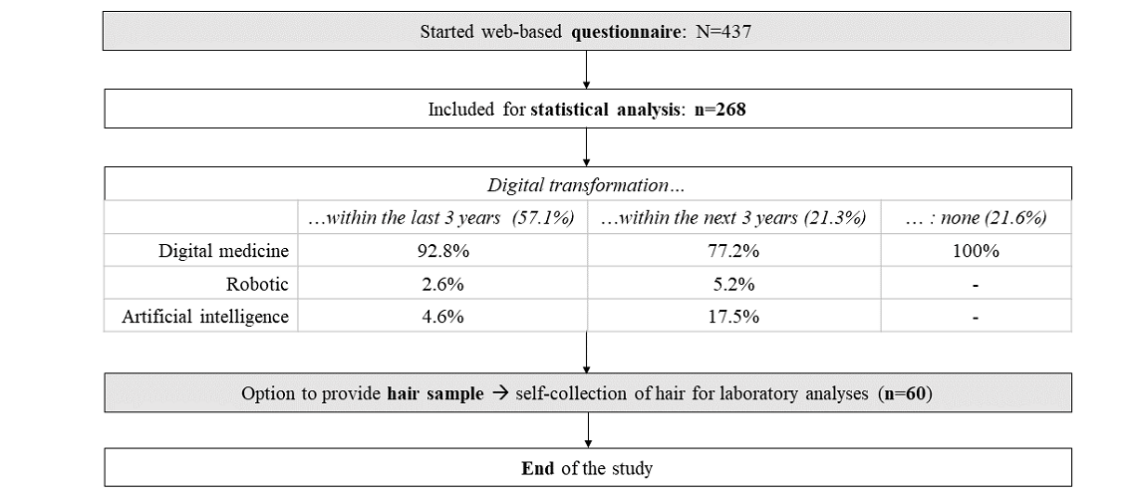
Participant flow through the study.

Overall, 57.1% (153/268) of the participants reported that a digital innovation was implemented at their workplace within the last 3 years, whereas 21.3% (57/268) of the individuals referred to a digital transformation that would be presumably implemented within the upcoming 3 years, and 21.6% (58/268) of the participants used our provided fictional example. Facing the large variety of different digital health care technologies mentioned by our study participants, we used three categories introduced by Topol [30] to categorize them: (1) *digital medicine* (ie, digital products and services that are intended for use in diagnosis, prevention, monitoring, and treatment such as EHRs and wearables), (2) *robotics* (ie, construction, operation, and application of intelligent machines, eg, robot-assisted surgery), and (3) *AI* (ie, methods that can be used to analyze, interpret, and make predictions using these data source, for example, AI-based diagnostics). Figure 1 presents the participant flow.

### Measures

This study had a cross-sectional, quantitative research design. The main part of the study was web-based questionnaire that was administered via LimeSurvey, encompassing questions regarding stress-relevant working conditions, sociodemographic factors (age, gender [men, women, nonbinary]), occupational characteristics (eg, specialization and years of work experiences), the actual implementation of a digital innovation at the workplace within the last 3 years (yes or no), and chronic stress. Sociodemographic factors and occupational characteristics were assessed using self-generated items. In addition, participants could send in self-collected hair samples as a biological marker of chronic stress.

Stress-relevant working conditions identified by Rau and Buyken [27] were assessed with the Short Questionnaire for Workplace Analysis (KFZA [31]). The KFZA seems especially suited as it fulfills the recommendations of the Joint German Occupational Safety and Health Strategy [32] for psychosocial workplace risk assessment and has been empirically proven to be generally applicable in hospital settings [33-36]. The KFZA originally consists of 26 items.

To take into account its applicability in a sample that is characterized by constant presence of time pressure, we shortened the KFZA to 11 items to enhance its applicability in physicians. Selection of items was based on the findings of Appel et al [33] as well as consensus of several experts. Within the original KFZA, the 26 items can be subsumed under 4 aspects of work. For each of these work aspects ≥1 items of the KFZA were included within this study: (1) “Job Content” (items in this study: *learning new skills*; *use of knowledge, skills, and abilities*; and *visibility of task accomplishment*), (2) “Resources” (items in this study: *influence on sequence of work activities and influence on workload and procedures*), (3) “Stressors” (items in this study: *workload, time pressure,* *excessive complexity of tasks,* *excessive demands on concentration,* and *interruptions to workflow*), and (4) “Organizational Culture” (item in this study: *consideration of employee input* [adjusted by referring to “clinic management” instead of originally referring to company management]). Items that are summarized under the work aspects 1, 2, and 4 have been theoretically and empirically associated with reduced work stress experiences (referred to as *work resources* in the following sections), whereas items that are summarized under work aspect 3 have been associated with enhanced stress experiences (referred to as *work stressors* in the following sections) [31,33]. Participants rated the extent to which they agree with each of these items on a 5-pont Likert scale, ranging from 1 (fully disagree) to 5 (fully agree). The internal consistencies (ie, Cronbach α) of the work aspects were calculated based on the current rating of the respective KFZA items (ie, preimplementation ratings of individuals without the actual implementation of a digital innovation and postimplementation ratings of individuals with the actual implementation of a digital innovation at one’s workplace) and ranged from acceptable to very good (work aspect 1: α=0.71, work aspect 2: α=0.89, and work aspect 3: α=0.77). No Cronbach α could be calculated for “Organizational Culture,” as this work factor was operationalized using only 1 item. As recommended by the authors of the KFZA, no sum scores over the different work aspects were calculated [31]. Instead, all items were analyzed separately.

Chronic self-reported stress was assessed using the German version [37] of the short form of the Perceived Stress Scale 4 (PSS-4 [38]). Respondents answered the 4 items on a 5-point Likert scale, ranging from 0 (never) to 4 (very often). Two items were reversed coded; thus, they were recoded before a total score was calculated, with higher values reflecting more stress (range 0 to 16). The internal consistency of the PSS-4 was, in accordance with the original publication [39], acceptable (Cronbach *α*=0.66).

Psychobiological indicators of stress were assessed via levels of the central hormone of the HPA axis, namely, cortisol, in the scalp hair. Cortisol mediates a number of biological, cognitive, and behavioral stress responses such as enhancing metabolic actions that increase energy level. These responses are necessary for adequately dealing with stressful situations and inhibiting stress-irrelevant body functions (eg, digestion [40]), making accumulated cortisol level a valid marker of HPA axis activity and therefore the chronic stress level of an individual [41]. In contrast to traditional measures of cortisol (blood, saliva, or urinary samples), the assessment of relevant HPA axis hormones in scalp hair retrospectively reflects an integrated secretion over several months [41], making hair F a valid and reliable biological marker of chronic stress [42]. Hair F concentration was determined from the 3 cm segment most proximal to the scalp. Given an average hair growth of 1 cm per month [43], this segment represents the cumulated cortisol secretion over a 3-month prior sampling episode. In the laboratory, handling and extraction of hair F were conducted in accordance with the laboratory protocol by Gao et al [44]. All samples were analyzed by liquid chromatography coupled with tandem mass spectrometry. The lower detection limit of the liquid chromatography coupled with tandem mass spectrometry protocol was 0.3 pg/mg for cortisol. All samples were processed in a single batch. The intraassay coefficients of variance was 8.2%.

### Ethical Considerations

The study was designed according to the ethical standards of the relevant national and institutional committees on human experimentation and with the Helsinki Declaration of 1975 (revised in 2008) and approved by the ethics committee of Technische Universität Dresden (identifier EK222052019). All participants provided informed consent. Data were pseudonymized during data collection using individually generated codes and anonymized after the data collection period.

### Procedure

After opening the link to the web-based questionnaire, participants were presented information about the study, and the participants provided informed consent. They were then asked about their medical specialty.

Next, participants read a short text, which explained the term digital transformation in medicine and included some examples. They were then asked to name and briefly describe a digital innovation at their workplace, which had either been implemented within the last 3 years or would be presumably implemented within the coming 3 years and which affected them personally and significantly altered their work. If no digital transformation matching these criteria existed, they were provided with a short description of the EHR, as EHRs are particularly widespread in all medical specialties. As depicted in Figure 1, most participants described digital health care technologies from the category of digital medicine (within the last 3 years: n=142, 53% and within the next 3 years: n=44, 16.4%); AI was the second most common category (within the last 3 years: n=7, 2.6% and within the next 3 years: n=10, 3.7%), ahead of robotics (within the last 3 years: n=4, 1.5% and within the next 3 years: n=3, 1.1%).

Thereafter, participants were asked to rate each of the KFZA items, regarding the situation both before and after the introduction of the respective digital transformation (exact wording: “Please rate how strongly the following statements apply to your work. As you do so, take turns imagining that the digital innovation you have just described does or does not affect you.”). In a final part of the web-based questionnaire, participants responded to items assessing perceived chronic psychological stress, sociodemographic variables, and further constructs, which are not part of this study.

Afterward, participants could receive information on how to self-collect hair samples, store them, and send them to the laboratory via mail. Previous research indicated that the self-collection of hair in domestic settings is a viable and economical method for measuring long-term steroid concentration in hair [45]. All individuals with hair >3 cm were invited to send in hair samples and were instructed to cut hair strains as close as possible to the scalp from the posterior vertex position.

As compensation, all participants were eligible to enter a lottery for 5 activity vouchers, each valued at €200 (US $215), and received personalized feedback on their chronic stress levels.

### Data Processing and Statistical Analyses

Hair F turned out to be positively skewed and was transformed on the natural logarithm scale to approach a normal distribution. For descriptive purposes, hair data in text and tables are reported in original units (pg/mg); however, for statistical analyses, log-transformed hair data were used.

First, we calculated descriptive statistics of study characteristics. Second, we examined potential differences in the chronic stress level between physicians from the most frequently mentioned medical specialties using an analysis of covariance (ANCOVA), with the PSS-4 as dependent and the medical specialty as independent variable, while adjusting for age and gender. We chose an ANCOVA as it allowed adjustment for the covariates gender and age. Due to the small number of hair samples per medical specialty, we abstained from calculating a respective ANCOVA for hair F. Third, we tested our first hypothesis that our selected KFZA items were stress relevant for physicians by investigating their associations with biopsychological markers of chronic stress. More precisely, we used nonparametrical partial correlation analyses controlling for age and gender to examine the association between the PSS-4, hair F, and the current rating of the respective KFZA items independent of any digital transformation. We opted for nonparametric partial correlation analyses to account for the different scale levels of the included variables. The ANCOVA and the nonparametrical partial correlation analyses were conducted using SPSS Statistics (version 28; IBM Corp).

Fourth, we tested our research hypotheses 2, 2.1, and 2.2 with multilevel linear mixed effects models nested within the person using the *nlme* function implemented in R (R Foundation for Statistical Computing) [46]. In all these models, time was included as within-subject variable, and age, gender, and the implementation of a digital innovation at one’s workplace within the last 3 years were included as level 2 predictors (model 1). To test our hypothesis 2 of differences in the rating of stress-relevant working conditions associated with digital transformation, separate multilevel linear mixed effects models were calculated for each KFZA item (dependent variable) to account for high collinearity (model 1). To test our hypothesis 2.1 on the influence of demographical variables on differences in the rating of stress-relevant working conditions associated with digital transformation, interaction terms for age and time as well as gender and time were added to model 1 (model 2). Here too, separate multilevel linear mixed effects models were calculated with each of the KFZA items serving as dependent variable. Hypothesis 2.2 on the influence of actual experiences with digital innovations at one’s workplace on differences in the rating of stress-relevant working conditions associated with digital transformation was tested by including an interaction term for this variable and time in model 2. We decided to use multilevel linear mixed effects models for testing our research hypotheses 2, 2.1, and 2.2 mainly because they provide a powerful method for examining complex data structures, which in our case enabled to differentiate between trait-like (intercepts) and digitalization-associated state-like (slopes) individual ratings of the KFZA items. In addition, multilevel linear mixed effects models allow for the consideration of covariates, and they provide better handling of missing values than most other common statistical methods.

For all analyses, we used the standard *P*<.05 criteria for determining if the results are significantly different from those expected if the null hypothesis were correct.

## Results

### Descriptive Results

In terms of chronic stress levels, these medical specialties significantly differed (*F*_5,167_=2.43; *P*=.04; η²=0.068), with highest perceived stress levels (PSS-4) in internal medicine (mean 7.03, SD 2.87) and the lowest in anesthesiology (mean 5.24, SD 3.14).

**Figure 2:**
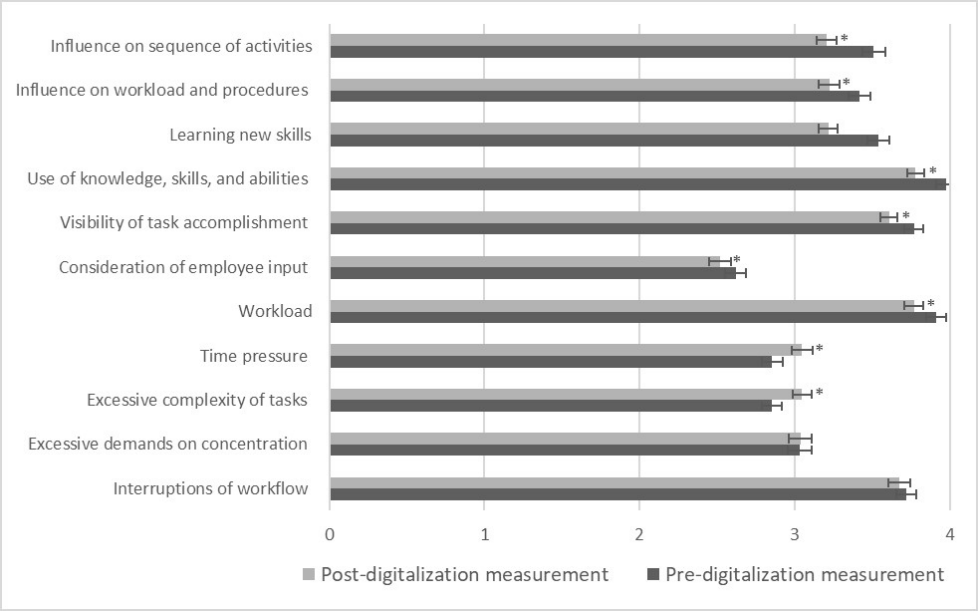
Mean ratings of stress-relevant working conditions operationalized using the Short Questionnaire for Workplace Analysis in the core sample. Error bars indicate SEs. *Indicates significant differences in the ratings before the digital transformation compared to after with *P*<.05 (for exact *P* values, refer to Tables 3 and 4).

An overview of the before and after ratings of all stress-relevant working conditions is given in Figure 2 [31]. Following the specifications made by Prümper et al [31], mean ratings >3 indicate a high perceived presence and mean ratings <3 indicate a low perceived presence of the respective working condition. Applying these criteria, the perceived working environment of the physicians in this sample is generally characterized by a relatively high amount of *interruption of their workflow*; a relatively high *perceived workload*; a relatively high *perceived visibility of task accomplishment*; and a relatively high perceived *use of knowledge, skills, and abilities*. In contrast, the physicians in the present sample perceived the *possibilities for their participation* in modification processes as rather low.

### Associations Between Chronic Stress and the Selected Working Conditions (H1)

To test our first hypothesis regarding the stress relevance of the selected working conditions, associations between the current rating of the KFZA items and chronic self-reported stress as well as hair F were evaluated. We operationalized the current ratings of KFZA items by comparing preimplementation ratings of individuals without the digital innovation and postimplementation ratings of individuals with the innovation in their workplace. These ratings reflect their current work situation. As expected, *work stressors* were positively associated with biopsychological markers of chronic stress, and *work resources* were negatively associated with biopsychological markers of chronic stress (Table 2). Thereby, chronic self-reported stress, operationalized using the PSS-4, was significantly associated with all working conditions, except for *visibility of task accomplishment*, which, however, only narrowly missed statistical significance (*P*=.05). Hair F depicted significant positive associations only with *excessive demands on concentration* and *consideration of employee input*. H1 can therefore be retained.

**Table 2:** Partial nonparametric correlations between markers of chronic stress and working conditions adjusting for age and gender.

Variable		PSS-4^a^ (N=268)	Hair F^b^ (n=60)
**Learning new skills**			
	*r*	−0.284	−0.113
	*P* value	<.001	.40
**Use of knowledge, skills, and abilities**			
	*r*	−0.193	−0.104
	*P* value	.002	.44
**Visibility of task accomplishment**			
	*r*	−0.117	−0.078
	*P* value	.06	.56
**Influence on sequence of work activities**			
	*r*	−0.206	0.076
	*P* value	.001	.57
**Influence on workload and procedures**			
	*r*	−0.222	0.095
	*P* value	<.001	.48
**Workload**			
	*r*	0.338	0.066
	*P* value	<.001	.62
**Time pressure**			
	*r*	0.261	0.029
	*P* value	<.001	.83
**Excessive complexity of tasks**			
	*r*	0.275	0.08
	*P* value	<.001	.55
**Excessive demands on concentration**			
	*r*	0.268	0.333
	*P* value	<.001	.01
**Interruptions to workflow**			
	*r*	0.241	0.134
	*P* value	<.001	.32
**Consideration of employee input**			
	*r*	−0.163	0.223
	*P* value	.008	.09

^a^PSS-4: Perceived Stress Scale 4.

^b^Hair F: hair cortisol concentration.

### Digitalization-Associated Differences in the Perception of Working Conditions (H2)

Our hypothesis 2 was that digital transformation would be associated with differences in the perception of stress-relevant working conditions, operationalized using the KFZA. Separate mixed linear effects model for each of the selected KFZA items were conducted. Results are depicted in Table 3 (*work resources*) and Table 4 (*work stressors*).

**Table 3:** Results from multilevel analysis on work resources (dependent variable) before and after digital transformations (within-subject variable) considering age, gender, and the implementation of a digital innovation at one’s workplace within the last 3 years.

			Stress-reducing working conditions (dependent variables), β coefficients (SE)					
			Influence on sequence of activities	Influence on workload and procedures	Learning new skills	Use of knowledge, skills, and abilities	Visibility of task accomplishment	Consideration of employee input
**Model 1**								
	Intercept		2.878 (0.244)**	2.756 (0.252)**	3.483 (0.244)**	3.487 (0.214)**	3.383 (0.205)**	3.051 (0.268)**
		Age	0.012 (0.005)*	0.012 (0.005)*	–0.004 (0.005)	0.010 (0.004)*	0.010 (0.004)*	–0.009 (0.006)
		Gender	0.086 (0.123)	0.146 (0.127)	0.152 (0.123)	–0.030 (0.108)	0.033 (0.103)	–0.254 (0.135)
		Impl dig^a^	0.184 (0.120)	0.132 (0.124)	0.217 (0.120)	0.202 (0.105)	–0.060 (0.101)	0.170 (0.132)
	**Within-subject variable**							
		Time	–0.302 (0.065)**	–0.194 (0.060)**	–0.093 (0.056)	–0.198 (0.057)**	–0.160 (0.055)**	-0.101 (0.042)*
**Model 2: Model 1+interactions**								
	Time×age		–0.021 (0.005)**	–0.022 (0.005)**	–0.012 (0.005)*	–0.016 (0.005)**	–0.012 (0.005)*	–0.008 (0.003)*
	Time×gender		0.181 (0.129)	0.145 (0.120)	0.035 (0.116)	–0.079 (0.116)	0.126 (0.113)	0.181 (0.085)*
	Time×impl dig		–0.112 (0.127)	–0.012 (0.118)	–0.182 (0.113)	–0.038 (0.113)	0.071 (0.110)	0.059 (0.083)
*χ*^2b^ (*df*)			23.8 (11)**	26.2 (11)**	11.2 (11)*	11.7 (11)**	9.9 (11)*	12.9 (11)**

^a^Impl dig: implementation of a digital innovation at one’s workplace within the last 3 years.

^b^χ² compares the lower level model with the respective next level model (ie, model 1 vs model 2).

*P<.05.

**P<.01.

**Table 4:** Results from multilevel analysis on work stressors (dependent variable) before and after digital transformations (within-subject variable) considering age, gender, and the implementation of a digital innovation at one’s workplace within the last 3 years.

			Stress-evoking working conditions (dependent variables), β coefficients (SE)				
			Workload	Time pressure	Excessive complexity of tasks	Excessive demands on concentration	Interruptions of workflow
**Model 1**							
	Intercept		3.975 (0.247)**	3.992 (0.238)**	3.402 (0.245)**	2.899 (0.293)**	4.889 (0.242)**
	**Control variables**						
		Age	–0.002 (0.005)	–0.002 (0.005)	–0.018 (0.005)**	0.001 (0.006)	–0.029 (0.005)**
		Gender	0.007 (0.125)	0.085 (0.120)	0.112 (0.123)	0.190 (0.148)	–0.056 (0.122)
		Impl dig^a^	0.058 (0.122)	0.148 (0.117)	0.203 (0.120)	-0.046 (0.145)	0.069 (0.119)
	**Within-subject variable**						
		Time	–0.146 (0.048)**	–0.175 (0.048)**	0.194 (0.062)**	0.004 (0.041)	–0.045 (0.054)
**Model 2: model 1+interactions**							
	Time×age		0.007 (0.004)	0.008 (0.004)*	0.016 (0.005)**	0.011 (0.003)**	0.014 (0.004)**
	Time×gender		–0.078 (0.098)	–0.100 (0.096)	0.042 (0.125)	–0.132 (0.083)	<0.001 (0.108)
	Time×impl dig		0.249 (0.096)**	0.436 (0.093)**	0.272 (0.123)*	0.038 (0.081)	0.330 (0.106)**
*χ* ^2b^			13.61 (11)**	31.43 (11)**	17.11 (11)**	17.77 (11)**	23.62 (11)**

^a^Impl dig: implementation of a digital innovation at one’s own workplace within the last 3 years.

^b^χ² compares the lower level model with the respective next level model (ie, model 1 vs model 2).

*P<.05.

**P<.01.

Significant main effects of time were revealed for 8 (72%) of the 11 KFZA items (refer to Table 3 for *work resources* and Table 4 for *work stressors*; model 2), which implies that H2 can, at least partly, be retained. For 6 (55%) of these 8 KFZA items for which significant main effects of time were revealed, digital transformation was associated with potentially stress-enhancing effects. More precisely, 5 *work resources* were rated lower (ie, *influence on sequence of work activities*: *t*_267_=−4.67; *P*<.001; *influence on workload and procedures:* *t*_267_=−3.21; *P*=.002; *use of knowledge, skills, and abilities:* *t*_267_=−3.49; *P*<.001; *visibility of task accomplishment:* *t*_267_=−2.92; *P*=.004; and *consideration of employee input:* *t*_267_=−2.41; *P*=.02), and one *work stressor* was rated higher (ie, *excessive complexity of tasks:* *t*_267_=3.14; *P*=.002) after the digital transformation compared to before. With regard to the KFZA item *consideration of employee input*, it should be noted that the effect does not stand up to a conservative Bonferroni correction (*P*<.005).

With respect to the 2 remaining KFZA items for which significant main effects were revealed, digital transformation was associated with potentially stress-reducing effects, as these *work stressors* (ie, *workload*: *t*_267_=−3.04; *P*=.003; *time pressure: t*_267_=−3.63; *P*<.001) were rated lower after the digital transformation compared to before. No significant main effects of time were revealed for the work resource *learning new skills* and the 2 work stressors *excessive demands on concentration* and *interruptions of workflow*.

### Influence of Age and Gender on Digitalization-Associated Differences in the Perception of Working Conditions (H2.1 and H2.2)

Results regarding our hypotheses H2.1 and H2.2 are depicted in Table 3 for *work resources* and in Table 4 for *work stressors* (model 3).

With respect to age, significant main effects on the rating of stress-relevant working conditions were revealed (model 1; Tables 3 and 4). More precisely, a higher age was associated with a less stressful perception of work, as indicated by higher ratings of *work resources* (ie, *influence on sequence of activities*; *influence on workload and procedures*; *use of knowledge, skills, and abilities*; and *visibility of task accomplishment*; model 1; Table 3) and lower ratings of *work stressors* (ie, *excessive complexity of tasks* and *interruptions of workflow*; model 1; Table 4).

**Figure 3:**
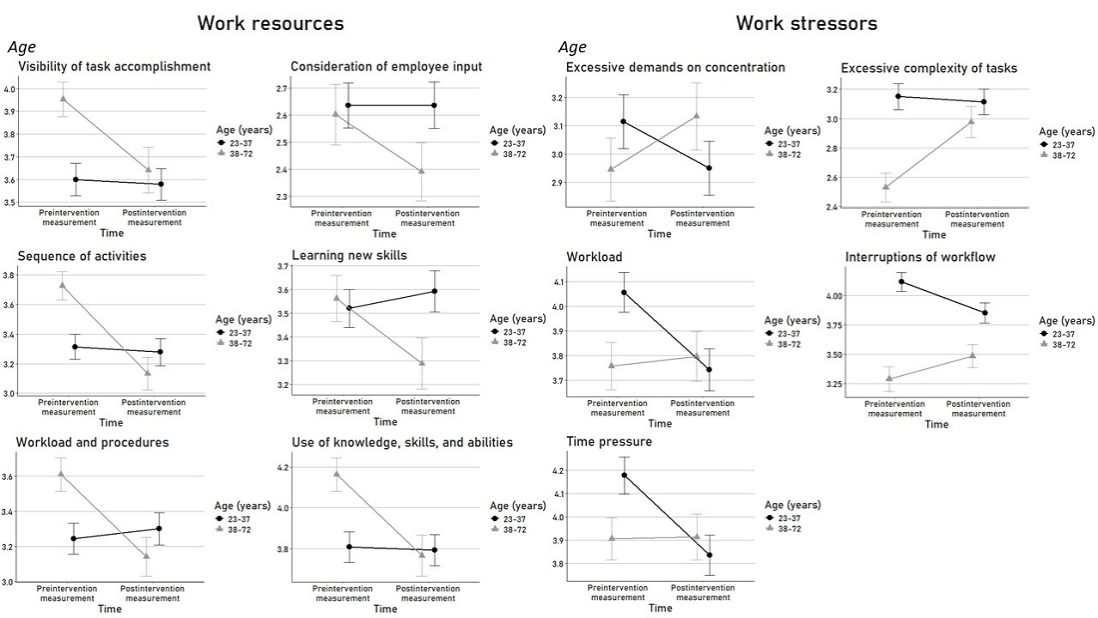
Significant interaction effects of time (before the digital transformation compared to after) and age on the perception of stress-relevant working conditions operationalized using the Short Questionnaire for Workplace Analysis.

The examination of our hypothesis H2.1 revealed significant interaction effects of time and age on all *work resources* and all *work stressors* (model 2; Tables 3 and 4). Thereby, a higher age was associated with lower ratings of *work resources* and higher ratings of *work stressors* after the digital transformation compared to before (Figure 3 [31]), indicating that older individuals rated differences associated with digital transformation in a stress-enhancing way compared to younger individuals.

No significant main effects of gender on ratings of stress-relevant working conditions were revealed (model 1; Tables 3 and 4).

**Figure 4:**
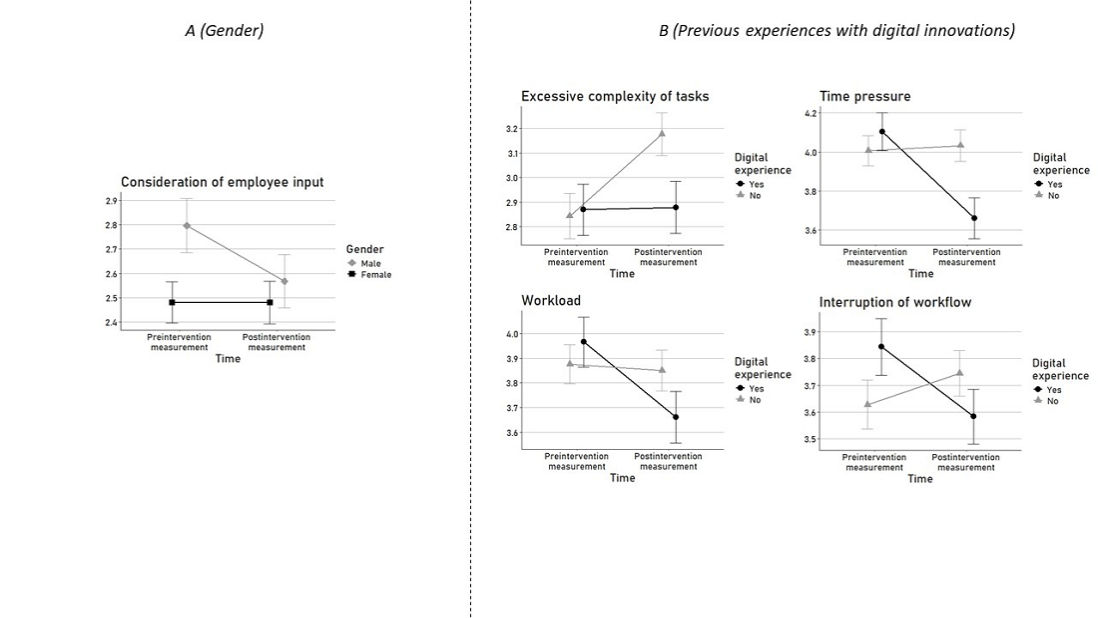
Significant interaction effects of time (before and after the digital transformation), gender (A), and previous experiences with digital innovations (B) on the perception of stress-relevant working conditions.

The testing of our hypothesis H2.1 revealed one significant interaction effect of time and gender on the rating of the KFZA item *consideration of employee input* (model 2; Tables 3 and 4). More precisely, compared to women, men rated this *work resource* lower after the digital transformation compared to before, indicating that men perceive differences associated with digital transformation in a stress-enhancing way (Figure 4 [31]).

In summary, it can be concluded that hypothesis 2.1, stating that age and gender influence the perceived differences in ratings of stress-relevant working conditions before and after the digital transformation, can be retained.

### Influence of the Actual Implementation of a Digital Innovation at One’s Workplace on Digitalization-Associated Differences in the Perception of Working Conditions

No main effect of the actual implementation of a digital innovation at one’s workplace within the last 3 years on the rating of stress-relevant working conditions was revealed (model 1; Tables 3 and 4).

The testing of our hypothesis H2.2 revealed significant interaction effects between time and implementation of a digital innovation at one’s workplace on the ratings of 4 *work stressors* (ie, *workload*, *time pressure*, *excessive complexity of tasks*, and *interruptions of workflow*; model 2; Table 4). On the basis of these results, our hypothesis H2.2 that the actual implementation of a digital innovation at one’s workplace is associated with differences in the perception of working conditions can be, at least with respect to 4 of the examined working conditions, retained. Thereby, individuals who actually experienced the implementation of a digital innovation at their workplace rated these *work stressors* higher post compared to pre digital transformation (Figure 4 [31]), indicating that actually experiencing the implementation of a digital innovation is associated with a rather stress-enhancing perception of digital transformation, compared to individuals who only imagined how these work stressors would differ in dependence of digital transformation.

## Discussion

### Principal Findings

The main goal of this study was to identify stress-related working conditions in physicians (H1), which are potentially prone to differ as a function of digital transformation in physicians (H2). In addition, we tested if these potential differences would be influenced by demographic variables (ie, age and gender; H2.1) and the actual experience of the implementation of a digital innovation at one’s workplace within the last 3 years (H2.2).

With respect to our first hypothesis, our results support, at least partly, the theoretically derived assumption that the selected working conditions (ie, KFZA items) were stress relevant for physicians, as 10 (90%) of the 11 KFZA items were significantly associated with either a psychological or a biological stress marker. Moreover, the direction of the revealed correlations confirms our categorization of the selected working conditions into *work resources* and *work stressors*. Our finding of associations with working conditions being found mainly with the psychological (PSS-4) and not with the biological (hair F) markers of chronic stress is consistent with a constantly revealed divergence between questionnaire-based measures and cortisol with respect to both hair F (for review, refer to the study by Stalder et al [29]) as well as other, more traditional cortisol measures, such as the cortisol awakening response [47], and cortisol (stress) reactivity [48,49]. Study-specific reasons for the lack of significant associations between the selected working conditions and hair F could be the reduced power in these analyses, as only a small number of participants could be included in these analyses, as well as shared method variance between the rating of the selected working conditions and the PSS-4.

In addition, our hypothesis H2 can be, at least partly, maintained, as our data suggest that digital transformation is associated with significant differences in the perception of 8 (73%) out of 11 stress-relevant working conditions. However, when interpreting these findings, it should be noted that one of these effects did not hold up to a Bonferroni corrector for multiple testing. Interestingly, the vast majority of these differences indicated a stress-enhancing effect of digital transformation (ie, enhanced *work stressors* and reduced *work resources* post compared to pre digital transformation). It should be noted, however, that we also found stress-reducing effects associated with digital transformation (ie, reduced *perceived workload* and reduced *time pressure*). In line with previous research [50,51], this finding contradicts single-sided views that describe processes of digital transformation at work either as an unambiguous savior from chronic stress or a fundamental negative occurrence with mainly negative effects. The stress-enhancing effects associated with digital transformation observed in this study were mainly conveyed by reductions in the perception of *work resources* post compared to pre digital transformation (ie, reduced *perceived own influence possibility on sequence of work activities*; lower *influence on workload and procedures*; less *use of knowledge, skills, and abilities*; less *visibility of task accomplishment*; and less *consideration of employee input*) and not by enhanced perception of *work stressors* (ie, only *excessive complexity of tasks* was rated higher post compared to pre digital transformation). This overall pattern is in line with previous research that suggested that digitalization-associated stress at work might rather be the result of a loss of resources than an increase in workplace stressors [13,16]. Moreover, our results reaffirm previous findings regarding the significance of resources in shaping how individuals perceive and cope with workplace stress [52,53].

Our findings provide important implications for the further development of theoretical models explaining the emergence of technostress [19,20]. They suggest that digital transformation is associated with differences in specific stress-relevant working conditions. Those working conditions should, if confirmed by larger longitudinal studies, be considered as potentially mediating variables. Furthermore, our data provide important insights on which working conditions to focus on for the health-promoting design of implementation processes of digital innovations. In this study, the types of digital transformation differed between participants. This suggests that very different types of digital transformations (ie, digital medicine, robotic, and AI) might result in similar differences in the perception of stress-relevant working conditions.

### The Role of Age and Gender

Our hypothesis 2.1 focused on the influence of central demographic variables on the perception of differences in stress-relevant working conditions associated with digital transformation.

Independent of digitalization, a higher age was associated with a general tendency to perceive working conditions in a less stressful way (ie, higher ratings of work resources and lower ratings of work stressors). Prior research on age-related work stress attributes this phenomenon to several factors. These include the progression of career development, with older workers typically occupying more favorable positions associated with lower chronic stress levels, as well as the development of enhanced stress coping skills over time. In addition, there may be a selection bias favoring stress-resistant employees, as individuals who perceive their work as stressful are more likely to withdraw from their jobs over the years compared to those who do not [54].

In terms of age-dependent differences in the perception of digital transformation at work, the results of this study indicate that older age was linked to a perception of working conditions as more stressful after the digital transformation compared to before. Finding age differences in technostress is in line with previous studies outside the medical context [55]. However, findings regarding the direction of this age effect are mixed, with some studies supporting our findings by revealing a positive association between an increasing age and an increasing technostress [56,57], whereas others showed the opposite pattern [21,58]. Several explanations for an age-dependent increase in technostress have been suggested, such as a diminishing acceptance and use of technology [59], a reduced ability to adapt to new technologies [60,61], less computer experience [55], less computer self-efficacy [55], or enhanced appraisal of difficulties to use digital applications [56,62] in older age.

With respect to gender, our findings indicate that men perceived differences in working conditions in a rather stress-enhancing way compared to women (ie, reduced rating of the work resource *consideration of employee input*). At first glance, this finding conflicts with the cultural tendency to understand technology as a masculine area [63], which would logically result in a more favorable perception of digital transformations for men compared to women. However, the findings of this study are in line with previous notions of women being more positive about the potential of digital transformation [64-66]. As the perception of digital transformation has been shown to influence the willingness to use these technologies [60], the results of this study might as well make an important contribution to accelerate the digitalization of the health system in a gender- and age-adapted manner.

### The Role of One’s Experience With Digital Innovations

With respect to our hypothesis 2.2, our results suggest that personal experiences with the implementation of digital innovations at one’s workplace resulted in a rather stress-enhancing evaluation of digital transformation (ie, higher ratings of 4 *work stressors* post compared to pre digital transformation) compared to only imagining these consequences. To the best of our knowledge, no previous study investigated differences between expected and experienced differences in the rating of stress-related working conditions. Therefore, further research is needed to evaluate the general validity of this finding. If confirmed, our finding suggests that, at least partly, the actual experiences with digital innovations was more negative than the expectations of physicians with no such experience, which would underline the need to evaluate the usefulness of digital innovations, preferably before they are introduced.

### Strengths and Limitations

The following limitations challenge the generalizability of the revealed results.

First, the cross-sectional design of this study (pre- and postdigital transformation ratings of the stress-related working conditions were collected at 1 time point only) does not allow to draw conclusions about the causality and the long-term stability of the revealed associations. The conclusion of the literature review by Berg-Beckhoff et al [50] that positive associations between digital technologies at work and stress being mostly found in cross-sectional studies instead of intervention studies underlines the importance of validating the revealed effects within methodically sound longitudinal field studies.

Second, to ensure the highest possible ecological validity, we chose not to focus on one single medical discipline and one specific type of digital transformation, which resulted in a relatively heterogeneous sample composition. The fact that significant effects were found even in such a heterogeneous sample basically speaks for the robustness of the revealed effects. One explanation for these cross-disciplinary effects could be that in everyday medical work the medical disciplines show a similar work distribution, workflows, and workload. For example, surgeons and urologists both operate using similar approaches (open, laparoscopic, and robotic-assisted surgery), both disciplines treat emergency patients additionally to elective cases, and both disciplines manage wards with inpatients. In addition, digital innovations such as the EHR are usually the same for all medical disciplines hospital wide. However, due to the small sample size in the respective conditions or subgroups, we were unable to examine stress-relevant effects of more specific types of digital transformation and specific medical disciplines. Future research with larger sample sizes is needed to examine possible differences in the effects with regard to these variables.

Third, data collection via a web-based survey might have influenced sample composition. More precisely, it seems plausible that the general attitude toward and familiarity with information technologies influences the willingness and capability to participate in a web-based survey, thereby limiting the generalizability of our results.

### Conclusions, Implications, and Outlook

Digital transformation processes are omnipresent and substantially alter work in medicine and physician-patient interactions. Therefore, it seems important to systematically evaluate the consequences that these processes may have for stress in physicians. In this regard, our study makes an important contribution by identifying those work stressors and resources that are potentially prone to differ between pre- and postdigital transformation in physicians, depending on age, gender, and previous experiences. If confirmed by comprehensive longitudinal studies, our results not only serve as a valuable addition to theoretical models elucidating the development of technostress in physicians but also carry significant practical implications for effectively navigating digital transformations. First, our findings hold concrete recommendations on which working conditions to focus on during design and implementation of digital innovation to reduce chronic stress in physicians. Moreover, our finding of a rather stress-enhancing effect of digital transformations emphasizes the importance of carefully evaluating in advance which digital innovations are truly beneficial for the user and which are more likely to deteriorate the working environment. Furthermore, our observation that this potentially stress-enhancing impact is largely driven by reductions in resources could imply the necessity for workplace interventions aimed at conserving resources during digital transformation processes and beyond. Finally, the age- and gender-dependent variations in the perception of digital transformation at work, revealed by this study, suggest that digitalization-associated training and support opportunities at work tailored to specific subgroups might be beneficial.

## Abbreviations

AI: artificial intelligence
ANCOVA: analysis of covariance
EHR: electronic health record
HPA: hypothalamus-pituitary-adrenal
KFZA: Short Questionnaire for Workplace Analysis
PSS-4: Perceived Stress Scale 4
